# Online mindfulness as a promising method to improve exercise capacity in heart disease: 12-month follow-up of a randomized controlled trial

**DOI:** 10.1371/journal.pone.0175923

**Published:** 2017-05-09

**Authors:** Rinske A. Gotink, John O. Younge, Machteld F. Wery, Elisabeth M. W. J. Utens, Michelle Michels, Dimitris Rizopoulos, Liesbeth F. C. van Rossum, Jolien W. Roos-Hesselink, Myriam M. G. Hunink

**Affiliations:** 1 Department of Epidemiology, Erasmus MC, Rotterdam, The Netherlands; 2 Department of Psychiatry, section Medical Psychology and Psychotherapy, Erasmus MC, Rotterdam, The Netherlands; 3 Department of Radiology, Erasmus MC, Rotterdam, The Netherlands; 4 Department of Cardiology, Erasmus MC, Rotterdam, The Netherlands; 5 Department of Child and Adolescent Psychiatry/Psychology, Erasmus MC, Rotterdam, The Netherlands; 6 Department of Biostatistics, Erasmus MC, Rotterdam, The Netherlands; 7 Department of Internal Medicine, Erasmus MC, Rotterdam, The Netherlands; 8 Centre for Health Decision Science, Harvard T.H. Chan School of Public Health, Boston, United States of America; Kurume University School of Medicine, JAPAN

## Abstract

There is increasing evidence that mindfulness can reduce stress, and thereby affect other psychological and physiological outcomes as well. Earlier, we reported the direct 3-month results of an online modified mindfulness-based stress reduction training in patients with heart disease, and now we evaluate the effect at 12-month follow-up. 324 patients (mean age 43.2 years, 53.7% male) were randomized in a 2:1 ratio to additional 3-month online mindfulness training or to usual care alone. The primary outcome was exercise capacity measured with the 6 minute walk test (6MWT). Secondary outcomes were blood pressure, heart rate, respiratory rate, NT-proBNP, cortisol levels (scalp hair sample), mental and physical functioning (SF-36), anxiety and depression (HADS), perceived stress (PSS), and social support (PSSS12). Differences between groups on the repeated outcome measures were analyzed with linear mixed models. At 12-months follow-up, participants showed a trend significant improvement exercise capacity (6MWT: 17.9 meters, p = 0.055) compared to UC. Cohen’s D showed significant but small improvement on exercise capacity (d = 0.22; 95%CI 0.05 to 0.39), systolic blood pressure (d = 0.19; 95%CI 0.03 to 0.36), mental functioning (d = 0.22; 95%CI 0.05 to 0.38) and depressive symptomatology (d = 0.18; 95%CI 0.02 to 0.35). All other outcome measures did not change statistically significantly. In the as-treated analysis, systolic blood pressure decreased significantly with 5.5 mmHg (p = 0.045; d = 0.23 (95%CI 0.05–0.41)). Online mindfulness training shows favorable albeit small long-term effects on exercise capacity, systolic blood pressure, mental functioning, and depressive symptomatology in patients with heart disease and might therefore be a beneficial addition to current clinical care.

**Trial registration:** www.trialregister.nl NTR3453

## Introduction

In recent decades, Mindfulness-Based Stress Reduction (MBSR) has grown to be a well-known adjunct intervention in Western healthcare with reproducible significant psychological improvements in multiple patient populations regarding depressive symptomatology, anxiety, stress, and quality of life [1]. Mindfulness is described as ‘the capacity to observe with open and non-judgmental awareness towards all experiences within the present moment’ [2]. Techniques taught as part of the eight-week MBSR training, mainly meditation, yoga and cognitive reappraisal, teach participants to be more present in the here and now and to be more aware of bodily sensations and internal psychological processes, which can increase the ability to recognize stress symptoms at an early stage. Stress from the mindfulness perspective refers to the tension that arises when we have negative experiences that we do not want [3] : MBSR teaches acceptance of negative emotions or thoughts as passing experiences and thereby reducing the stress associated with them [4]. People with chronic conditions are prone to having negative thoughts and feelings they do not want (depression and anxiety comorbidity is high [5, 6]) and MBSR has been found to positively affect psychological outcomes in patients with chronic pain, obesity, hypertension, depression, anxiety and cardiovascular disease [7–11]. Over one million people in the Netherlands suffer from cardiovascular disease, and each year 100.000 get diagnosed. Healthcare costs are eight billion euro; 9.2% of total healthcare costs [12]. Cardiovascular disease is affected by stress: high perceived stress is associated with a risk ratio of 1.27 for incident coronary heart disease [13] , presence of psychosocial stressors is associated with increased risk of acute myocardial infarction [14] and it negatively affects heart rate, blood pressure and inflammatory factors [15]. On the contrary, low and variable heart rate and low blood pressure are associated with long-term survival and according to the ESC Guidelines cardiovascular patients are recommended to reduce stress in order to favorably affect these risk factors [16].

MBSR has shown to improve heart rate, breathing patterns and blood pressure in cardiovascular patients [17, 18]. Lower blood pressure and heart rate are directly related to exercise capacity [19–21] and a walking distance of <300 meters on the six minute walking test is a prognostic marker of subsequent cardiac death in patients with mild to moderate congestive heart failure [22]. The rationale of this randomized controlled trial is that in reducing stress, mindfulness therapy might influence heart rate, breathing patterns and blood pressure. These physiological effects may in turn improve exercise capacity and thus long-term outcome in cardiovascular patients [23]. In 3-month post-intervention follow-up, participants who received an online mindfulness training showed a higher mean distance on the 6-minute walk test, however this was small and borderline statistically significant (13.4 metres, p = 0.050) [24]. This article reports the results of the 12-month follow-up.

## Materials and methods

### Study design

The current study is a single blinded, pragmatic RCT performed at the outpatient cardiology clinic of the Erasmus MC, Rotterdam, the Netherlands. Detailed description of design and methodology, and 3-month results have been reported elsewhere [24]. Ethical approval was obtained from the Medical Ethics Committee of the Erasmus Medical Center and the study complies with the Declaration of Helsinki. The study was registered with the Dutch trial registry, NTR3453, http://www.trialregister.nl.

### Participants

Adult patients, between 18 and 65 years of age, with existing diagnosed heart disease (ischemic, valvular, congenital heart disease, or cardiomyopathy) were approached between June 2012 and April 2014 during their scheduled yearly visit at the outpatient clinic. Patients were excluded when there was: (1) a planned operation or percutaneous intervention within the upcoming year; (2) inability to understand, read, or write Dutch; (3) no internet access, email, or cell phone. After written informed consent was obtained and baseline measurements were performed, patients were randomized according to a 2:1 ratio to the intervention or control group via dedicated computer software (ALEA^®^) with a block size of 12 [25].

### Intervention

The mindfulness training consisted of a 12-week structured online program (see Table A in S1 File), which was offered in addition to usual care (UC) as provided by the treating cardiologist. Participants also received a book about mindfulness by a renowned author to support the 12-week training [26]. The intervention started as soon as patients logged in on the mindfulness training website, to which they gained access the day of the inclusion. Online delivery of the training was chosen for pragmatic reasons: the training was designed to be self-directed, easily accessible and engaging to a wide audience by keeping practice sessions and lessons short, usually ten to fifteen minutes per exercise. The program teaches different meditations, self-reflection, yoga exercises, and includes practical assignments and suggestions for mindfulness in daily life. The use of the breath as a reminder for present moment awareness is emphasized in all meditations. During the course participants also received biweekly reminders by e-mail and standardized text messages. After the 12-week online intervention, these reminders continued until the 12 month follow-up. Adherence was monitored by whether the questions of the online program were completed, without disclosing the content of the answers. The control group received UC by their treating cardiologist. We considered any partial placebo effect an integral part of the active intervention as it would be when implemented in day-to-day practice.

### Outcome measures

Outcomes were measured in all patients at baseline, post-intervention (3 months), and 9 months after the intervention was completed (12 months). Blinding of patients was not possible due to the nature of the intervention, but the outcome assessors were unaware of patients’ treatment allocation, and patients were instructed not to disclose their treatment allocation to the study investigators nor to their cardiologist.

To measure exercise capacity, the 6 Minute Walking Test (6MWT) was chosen as primary outcome measure, performed in a quiet corridor of the outpatient clinic [27]. Patients were instructed to walk the greatest distance they could in 6 minutes. Secondary outcome measures were physical parameters (blood pressure, respiratory rate, and heart rate), blood sampling laboratory test (N-terminal pro-brain natriuretic peptide (NT-proBNP) measured from peripheral venous blood samples), and hair cortisol as a biomarker of stress using ELISA as previously described [28]. Details on lab procedures can be found in the 3-month article [24].

To assess psychological functioning, we measured quality of life (using the Short-Form Health survey 36 [29] and a Visual Analogue Scale ranging from 0 to 100 [30]), anxiety and depression (Hospital Anxiety and Depression scale [31]), perceived stress (Perceived Stress Scale [32]) and social support (Perceived Social Support Scale (PSSS12)[33]). The use of other complementary care was monitored with a questionnaire (type, frequency, and intensity).

### Quality control and statistical analyses

An independent audit was performed and the study was found to comply with Good Clinical Practice and Scientific Integrity standards.

To demonstrate an improvement of 5% in the intervention group vs 1% in the control group on the 6MWT, this study required 99 patients in the control group and 198 in the active intervention group (SD10%, alpha = 0.05, power = 0.90, ratio experimental to controls = 2). Even if only 50% of patients in the experimental group adhered to the training, this would give us a power of 0.80 in the as-treated analysis. To account for non-adherence and loss to follow-up our aim was to randomize at least 300 patients. This number of patients is sufficient to demonstrate a smaller difference (5% in the intervention group vs 2% in the control group) in a repeated measurements analysis with a power of 75% (2 follow-up measurements, correlation between follow up measurements = 0.70, correlation between baseline & follow-up = 0.50).

Changes in outcomes at 12 months were compared with baseline and between treatment groups. An intention-to-treat (ITT) analysis was performed to address whether offering a mindfulness training was effective compared to UC. An as-treated (AT) analysis was performed to address whether the mindfulness training was beneficial if actually performed. In the AT analysis, patients were considered adherent if they completed 50% or more of the exercises. Patients allocated to the UC group who sought mindfulness training on their own initiative were excluded from the AT analysis.

Repeated measurements analyses using a multivariate linear regression mixed model were performed to determine intergroup effects and to simultaneously account for the correlation between the repeated measurements of each patient and for missing values. In the mean structure of the mixed model we included the time effect, the intervention effect and their interaction, while a fully unstructured variance-covariance matrix was assumed for the error terms. Due to randomization only p-values for the interaction effect are reported. In order to compare the different outcome measures, Cohen’s d was calculated based on the linear mixed model results. Finally, we performed log linear regression analyses to see which participants were most likely to adhere to the training, and if adherent, what characteristics predicted the most benefit from the training. P<0.05 was considered to be indicative of statistical significance. All data were analyzed with SPSS version 21.0 [34].

## Results and discussion

### Patient characteristics

**Fig 1:**
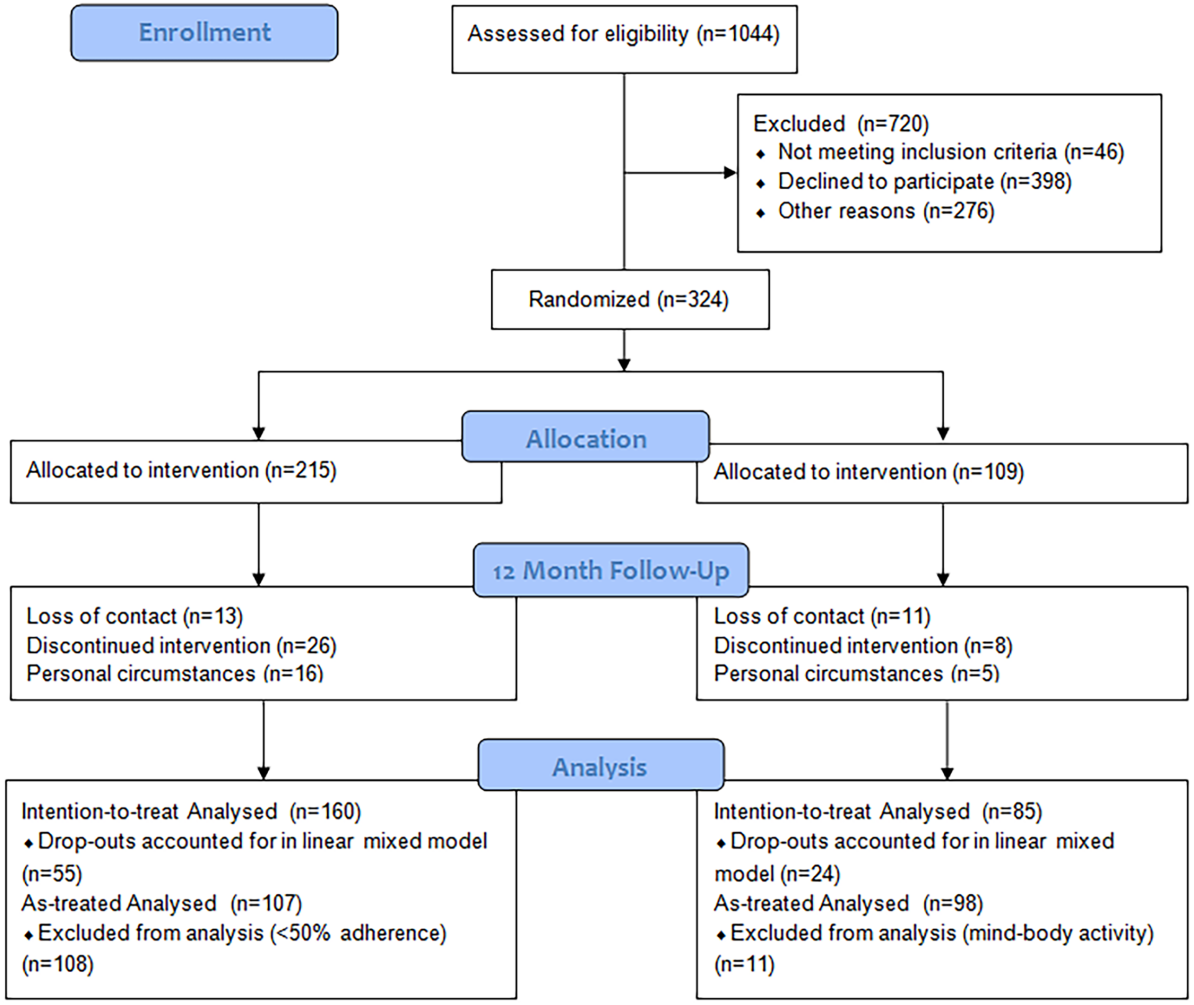
Flowchart of mindfulness intervention group and control group. *Linear mixed effects models use all available data and obtains valid inferences under the missing at random assumption.

Fig 1 displays the flowchart of the patients’ recruitment and follow-up. Table 1 shows participants’ baseline characteristics. A total of 324 patients were included and successfully randomized over the two treatment arms. Of the initial study population, 245 participants returned for long-term follow-up (75.6%), and 224 participants were present at all three measurement moments. No significant differences were found between the groups at follow-up with regard to demographic and clinical variables, and this percentage of follow-up still gives us sufficient power and the assumptions of our statistical tests were met. No major side effects were reported during the follow-up period.

**Table 1:** Baseline characteristics of study participants.

	Mindfulness Group N = 215	Control Group N = 109
Demographics		
Age (years), mean (SD)	43.2 (14.1)	43.2 (13.7)
Female (%)	44.2	50.5
Employed (%)	68.7	67.9
Body mass index (kg/m^2^), mean (SD)	25.9 (4.6)	25.7 (4.7)
Cardiac history		
Type of heart disease, (%)		
Congenital heart disease ^a^	41.9	42.2
Cardiomyopathy	39.5	29.4
Valvular heart disease	18.6	28.4
Number of interventions ^b^ mean (SD)	1.4 (1.4)	1.4 (1.2)
Time since first intervention (years) mean (SD)	19.1 (14.0)	15.9 (11.7)
Implantable cardioverter-defibrillator (%)	5.9	4.3
Pacemaker (%)	9.3	5.2
Intoxication, (%)		
Current smoking	14.4	18.3
Current alcohol use	62.1	55.0
Current drugs use	3.3	2.8
Prior use of complementary therapies ^c^ (%)	14.4	12.8

SD: Standard deviation

^a^ Tetralogy of Fallot, transposition of the great arteries, Fontan-circulation, coarctation of the aorta, and Ebstein’s disease

^b^ Includes both surgical and percutaneous interventions

^c^ Includes yoga, meditation, mindfulness, Tai Chi, Qigong and acupuncture

### Outcome analysis

In the ITT analyses, the mindfulness group showed an improvement of 17.9 meters on their mean 6MWT at 12 months compared to UC, which was not statistically significant (p = 0.055) (Table 2). Heart rate, systolic and diastolic blood pressure, and hair cortisol level decreased over time, but not significantly different from UC. Analyses on psychological outcomes showed no significant differences between the groups. Anxiety, depression and stress levels decreased stronger in the mindfulness group than in UC, but not statistically significantly.

**Table 2:** Outcomes at baseline and 12 months, and linear mixed models-based estimated difference (β) of intervention group compared to control over time.

**Intention-to-treat analysis.**						
O**utcome**	**Treatment group**	**Baseline (mean, SD)** **N = 324**	**12 months (mean, SD)** **N = 245**	**Difference (β)**	**95% Confidence Interval**	**p-value**
6MWT, meters	Mindfulness	537.5 (77.0)	549.0 (81.6)	+17.9	-0.4 to 36.2	0.055
	UC	539.3 (67.3)	532.9 (82.8)			
Heart rate, beats/min	Mindfulness	68 (12)	67 (12)	-0.2	-3.2 to 2.8	0.897
	UC	69 (11)	68 (12)			
SBP, mmHg	Mindfulness	127.5 (16)	123.8 (17)	-3.8	-8.2 to 0.5	0.085
	UC	125.4 (15)	125.4 (17)			
DBP, mmHg	Mindfulness	78.0 (11)	77.0 (10)	+1.5	-1.0 to 4.1	0.240
	UC	79.7 (10)	77.1 (10)			
NT-proBNP, pmol/L°	Mindfulness	2.9 (1.2)	2.9 (1.3)	+0.01	-0.2 to 0.2	0.902
	UC	3.0 (1.2)	3.0 (1.2)			
Cortisol (Hair pg/mg)	Mindfulness	35.8 (145.4)	32.0 (34.2)	+6.5	-18.9 to 31.8	0.614
	UC	40.2 (199.6)	30.0 (45.2)			
Physical QoL (SF-36)	Mindfulness	46.7 (9.6)	46.3 (9.2)	-1.6	-3.4 to 0.3	0.091
	UC	45.3 (10.3)	46.4 (9.4)			
Mental QoL (SF-36)	Mindfulness	50.1 (10.6)	51.6 (10.5)	+2.2	-0.5 to 4.8	0.108
	UC	50.8 (9.6)	50.1 (10.5)			
Quality of life (VAS)	Mindfulness	75.0 (13.2)	75.5 (12.0)	-1.8	-4.9 to 1.4	0.265
	UC	72.5 (13.2)	74.8 (12.2)			
Anxiety (HADS)	Mindfulness	8.2 (3.6)	7.5 (3.6)	+0.7	-0.2 to 1.5	0.156
	UC	9.0 (3.4)	7.6 (3.6)			
Depression (HADS)	Mindfulness	3.8 (2.9)	3.3 (2.7)	-0.5	-1.2 to 0.2	0.143
	UC	3.8 (2.9)	3.8 (2.7)			
Stress (PSS)	Mindfulness	22.4 (7.8)	20.2 (8.1)	-1.4	-3.4 to 0.7	0.189
	UC	22.0 (7.5)	21.1 (8.2)			
Social support (PSSS12)	Mindfulness	69.5 (11.6)	70.7 (12.4)	+1.7	-1.3 to 4.6	0.262
	UC	71.2 (12.3)	70.7 (12.5)			
**As-treated analysis.**						
O**utcome**	**Treatment group**	**Baseline (mean, SD)** **N = 205**	**12 months (mean, SD)** **N = 205**	**Difference (β)**	**95% Confidence Interval**	**p-value**
6MWT, meters	Mindfulness	532.6 (96.9)	541.5 (139.6)	+16.5	-6.2 to 39.3	0.153
	UC	538.2 (101.3)	530.6 (148.5)			
Heart rate, beats/min	Mindfulness	68.4 (16.6)	67.8 (18.4)	+1.0	-2.6 to 4.6	0.582
	UC	68.9 (17.3)	67.3 (19.7)			
SBP, mmHg	Mindfulness	129.7(22.8)	124.4 (27.3)	-5.5*	-10.9 to -0.1	0.045
	UC	125.8(23.8)	126.1 (29.2)			
DBP, mmHg	Mindfulness	79.4 (15.4)	77.8 (16.3)	+0.6	-2.5 to 3.7	0.687
	UC	79.9 (16.1)	77.6 (17.4)			
NT-proBNP, pmol/L°	Mindfulness	3.0 (1.4)	3.1 (1.4)	+0.07	-0.2 to 0.3	0.527
	UC	2.9 (1.4)	3.0 (1.4)			
Cortisol (Hair pg/mg)	Mindfulness	41.8 (165.0)	31.4 (41.7)	+1.6	-31.2 to 34.4	0.924
	UC	41.9 (194.8)	29.9 (45.1)			
Physical QoL (SF-36)	Mindfulness	45.7 (13.6)	45.2 (15.0)	-1.9	-4.1 to 0.2	0.081
	UC	45.4 (14.2)	46.9 (15.9)			
Mental QoL (SF-36)	Mindfulness	49.8 (13.5)	50.8 (17.0)	+2.3	-0.6 to 5.3	0.119
	UC	51.7 (14.1)	50.0 (18.2)			
Quality of life (VAS)	Mindfulness	74.5 (18.4)	74.6 (18.8)	-1.9	-5.5 to 1.6	0.288
	UC	73.4 (19.3)	75.4 (20.1)			
Anxiety (HADS)	Mindfulness	8.3 (4.8)	7.6 (5.4)	+0.6	-0.4 to 1.5	0.248
	UC	9.0 (5.0)	7.8 (5.8)			
Depression (HADS)	Mindfulness	3.8 (4.0)	3.2 (4.4)	-0.7	-1.5 to 0.1	0.100
	UC	3.6 (4.2)	3.7 (4.7)			
Stress (PSS)	Mindfulness	22.4 (10.5)	20.5 (12.4)	-1.3	-3.6 to 1.0	0.275
	UC	21.8 (11.0)	21.2 (13.3)			
Social support (PSSS12)	Mindfulness	69.2 (16.7)	70.0 (19.9)	+1.2	-2.5 to 4.9	0.522
	UC	71.9 (17.4)	71.6 (21.5)			

Outcomes at baseline and 12 months, and Linear Mixed Models-based estimated difference (β) of intervention group compared to control over time. SD, standard deviation; SE, standard error; 6MWT, six-minute walk test; UC, usual care; SBP, systolic blood pressure; DBP, diastolic blood pressure; NT-proBNP, N-terminal pro-brain natriuretic peptide; SF-36, Short Form Health survey; QoL, Quality of Life; VAS, visual analogue scale; HADS, hospital anxiety and depression scale; PSS, perceived stress score; PSSS12, perceived social support scale

° log-transformed,

* Significant at p<0.05

In the AT analyses (Table 2), 205 participants (63.3%) were adherent to their allocated group: in the intervention group 49.8% (N = 107) completed at least 50% of the training, and in the control group 89.9% (N = 98) performed no mind-body practice. Systolic blood pressure decreased significantly with 5.5 mmHg (p = 0.045) compared to UC. The other outcomes were similar to the ITT analysis.

### Standardized effect Size

**Fig 2:**
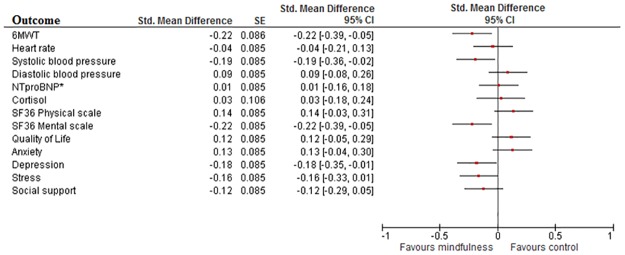
Cohen’s D in intention-to-treat analysis. Plot showing Cohen’s D effect measures of online mindfulness compared to treatment as usual in the Intention-To-Treat analysis. All values lower than 0 indicate a significant difference in favour of mindfulness. The breadth of the line indicates the 95%CI. Values between 0 and -0.2 indicate negligible effect; between -0.2 and -0.5 small effect; between -0.5 and -0.8 medium effect and lower than -0.8 a large effect. *: log transformed values.

**Fig 3:**
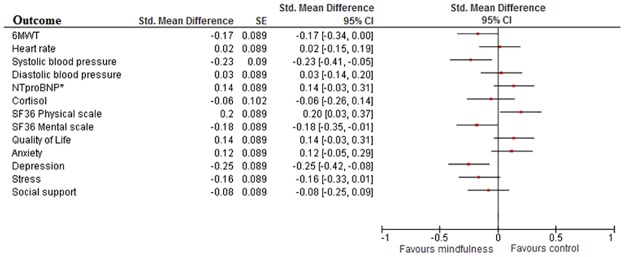
Cohen’s D in as-treated analysis. Plot showing Cohen’s D effect measures of online mindfulness compared to treatment as usual in the As-Treated analysis. All values lower than 0 indicate a significant difference in favour of mindfulness. The breadth of the line indicates the 95%CI. Values between 0 and -0.2 indicate negligible effect; between -0.2 and -0.5 small effect; between -0.5 and -0.8 medium effect and lower than -0.8 a large effect. *: log transformed values.

Cohen’s D calculation of outcome measures resulted in significant improvements on the 6MWT (d = 0.22, 95%CI 0.05 to 0.39), systolic blood pressure (d = 0.19, 95%CI 0.03 to 0.36), mental functioning (d = 0.22, 95%CI 0.05 to 0.38) and depression (d = 0.18, 95%CI 0.02 to 0.35) compared to UC. All other outcomes showed no significant differences (Fig 2). Similar though smaller effects were found in the as-treated analyses (Fig 3).

### Effect of compliance

Regression modelling of adherence showed that women (β = 0.86, p = 0.045), and with a higher diastolic blood pressure (β = 0.04 mmHg, p = 0.031) are more often compliant (Table B in S1 File). However when compliant to the online training, men (β = -23.1, p = 0.015) with a lower BMI (β = -2.1 kg/m, p = 0.048) improve more on the 6MWT. Also having higher stress levels (PSS β = 2.6, p = 0.007) and experiencing little mental hindrances (MCS β = 1.7, p = 0.011) are associated with a better effect of the training on the 6MWT (Table C in S1 File).

### Discussion

**Fig 4:**
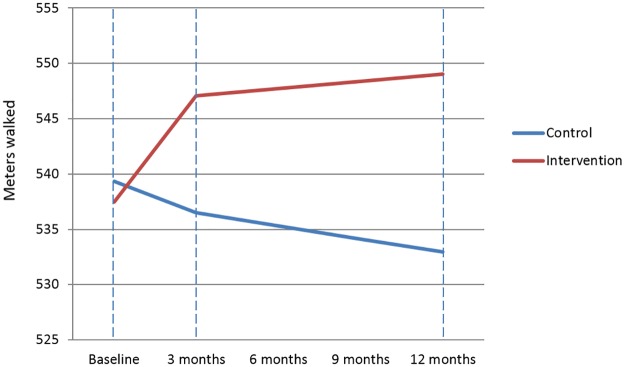
Linear mixed models results. Plot showing Linear Mixed Models results: the mean distance walked in meters by the Intervention group (red) and the Control group (blue) at each of the three measurement moments.

To our knowledge, this is the first randomized trial to evaluate the long-term effectiveness of an online mindfulness training on physical fitness in patients with heart disease. Our rationale was that by improving stress-related cardiovascular risk factors, mindfulness could improve physical functioning in these patients. On the primary endpoint we found that the original improvement of 13.4 meters (p = 0.050) measured directly after the online training was extended to 17.9 meters (p = 0.055) in favor of the mindfulness group (Fig 4). Using Cohen’s D (which is based on a Z-distribution, where mixed models uses a T-distribution), exercise capacity, systolic blood pressure, mental functioning, and depression improved significantly compared to UC. This shows how choice of statistical method can make a difference in conclusions, especially when p-values are close to the significance level. While 17.9 meters with a d = 0.22 is a small effect, it still gives an indication of potential long-term health benefit for patients with heart disease by using mindfulness.

There are several limitations to take into account. It could be that in our aim to construct a pragmatic and easy-accessible training, the working components of the MBSR protocol were cut too short, as our sample size was sufficient and the randomization procedure succeeded. We anticipated a 50% dropout in our 2:1 randomization ratio, which proved exactly right (49.8% of participants allocated to online mindfulness adhered to the training). Furthermore, the online training was low in intensity and our hypothesis concerned a two-stage effect of a psychological intervention on physical fitness. Regarding the level of statistical significance, a slight increase in training intensity could strengthen our results, as there is a large difference in dose compared to the full MBSR protocol. Also, our patients’ psychological baseline scores were similar to scores in the general population [35–39] which could explain the abstinence of improvements due to a ceiling effect. Similarly, our participants’ blood pressure was monitored regularly by the outpatient clinic and medication was given if necessary, resulting in fairly normal baseline values and little room for improvement. Other studies showing effects on either psychological symptoms or on blood pressure, investigated populations whose values at baseline were higher than average [17, 40]. Three other studies on web-based mindfulness training showed that it is feasible to conduct online mindfulness training, and also that it was effective in reducing stress [41–43]. Due to limited power for sub-group analyses, we have to be careful drawing firm conclusions, but results indicate that, although older women with a higher diastolic blood pressure are generally more compliant to this type of intervention, they appear to benefit less. This could be taken into account as well in intensifying the future online program.

The training was expected to have less effect than MBSR due to its lower intensity, but it also lacked other aspects: there was no social interaction nor any form of feedback. As there was no social control, it was completely left to participants whether they practiced or not. This can lead to less motivation and lower adherence than a training with teacher and other group members. The current online training may therefore have been too ‘light’ and too far withdrawn from the original MBSR. While this would mean that MBSR may have stronger effects, the accessibility of online training possibly allows better generalizability of the results, as patients can do the training in their own environment and fit it into their schedule. A middle way would therefore be ideal: an easily accessible online training, but with more content and feedback from a trainer. Additionally, the control group was aware that they were not receiving the online mindfulness training. Finally, we did not measure mindfulness skills, so we cannot confirm that changes are correlated with improvement of mindfulness skills. Although the only difference between the randomized groups was the online training, it would add confirmation if future studies also include this outcome.

## Conclusions

Online mindfulness training shows promising long-term effects on exercise capacity in patients with heart disease, but further research is necessary.

## Supporting information

S1 Document Study protocol. (PDF)

S2 Document Consort checklist. (DOC)

S1 File Containing: Table 1. Content of online training compared to standardized 8-week MBSR protocol. Table 2. Regression model of training adherence (N = 215). Table 3. Regression model of improvement on 6MWT when adherent (N = 107).
(DOCX)
