# Supplementary material for: Online mindfulness as a promising method to improve exercise capacity in heart disease: 12-month follow-up of a randomized controlled trial
Source: PLoS One. 2017 May 9;12(5):e0175923. doi: 10.1371/journal.pone.0175923 (PMC5423609; doi:10.1371/journal.pone.0175923)
Supplement: S1 File — Containing: Table 1. Content of online training compared to standardized 8-week MBSR protocol. Table 2. Regression model of training adherence (N = 215). Table 3. Regression model of improvement on 6MWT when adherent (N = 107). (DOCX) [file pone.0175923.s003.docx]

**Suplementary S1 File**

Contains:

Table 1. Content of online training compared to standardized 8-week MBSR protocol.

Table 2. Regression model of training adherence (N=215)

Table 3. Regression model of improvement on 6MWT when adherent (N=107).

**Table A.** Content of online training compared to standardized 8-week MBSR protocol.

| **Week** | **MBSR** | | | **Online training** | | |
| --- | --- | --- | --- | --- | --- | --- |
| **1** | **Automatic pilot** | | | **Do you really feel alive, don’t watch but see** | | |
|  | - Introduction to mindfulness - Raisin exercise - 40 minutes Bodyscan - Working of the breath - Automatic pilot | | | - Introduction film Edel Maex - Likert-scale ‘really living’ - Exercise focused attention, watch your computer - 15 minutes Bodyscan - Observing pictures - What deserves more attention in your life? | | |
| **Home-work** | - Daily 40 min Bodyscan - Daily mindful eating - 3x a day aware breathing - Daily journal keeping - Puzzle | | | - Weekly 15 min Bodyscan | | |
| **2** | **Supporting factors** | | | | | **Hear what people say, avoid the automatic pilot** |
|  | - 40 min Bodyscan - 8 supporting factors - Plan your practices - Pain, disease and attention - Thoughts, feelings and bodily sensations | | | | | - Adjust questioning and listening behavior - Short film on living separated from your surroundings - List of daily activities performed on automatic pilot |
| **Home-work** | - Daily 40 min Bodyscan - Daily 10 min. Sitting meditation - Daily routine-activity - Calendar of pleasant events - 3x per day 3-minutes breathing space | | | | | - Execute 3 automatic activities with mindfulness |
| **3** | **Bodily experiences** | | | | **Learn to meditate** | |
|  | - 25 min sitting meditation - 45 min Yoga - Boundaries of the body - Movement with health issues | | | | - Short film Edel Maex - 15 min sitting meditation - Planning sitting meditation - Remember what deserves more attention? | |
| **Home-work** | - Daily Bodyscan or Yoga (40 min) - Daily 15 min attention training - Calendar of unpleasant events - Noticing the automatic pilot | | | | - Daily 15 min sitting meditation | |
| **4** | **Stress** | | | | **Dealing with stress, today is an important day** | |
|  | - 40 min sitting meditation - 25 min Yoga - Stress reaction and symptoms - Reaction on unpleasant events - Resistance or avoidance - Stress aware responding - 3-minute breathing space | | | | - Short film stress reaction - 3-minute breathing space - Description of current day | |
| **Home-work** | - Daily Bodyscan or Yoga (40 min) - Daily 20 min sitting meditation - Calendar of stressful events - 3x per day 3-minute breathing space | | | | |  |
| **5** | **Habits and change** | | | | | **Learn to stop aware, your own mindfulness-reminder** |
|  | - 40 min sitting meditation - Habits and patterns - Our self-healing abilities - Change begins with awareness - 10 min walking meditation | | | | | - Inventory stopping with current activity - Short film with quotes - Short film about death |
| **Home-work** | - Daily sitting meditation or Yoga (40 min) - 3x per day 3-minute breathing space - Calendar of stressful conversations - Daily activity with awareness | | | | | - 3x per day 20 minutes stopping current activity; with awareness |
| **6** | **Thoughts and senses** | | | | | **You are not your thoughts** |
|  | - 40 min sitting meditation - Approach without judgment - Experience the senses - 20 min walking meditation | | | | | - Thoughts are not facts, you are not your thoughts - Difficult memories - Being compassionate |
| **Home-work** | - Daily 40 min sitting meditation, Yoga or Bodyscan - Integrating mindfulness in conversations - Noticing sensory experiences - Noticing the automatic pilot | | | | |  |
| **7** | **Feelings** | | | | | **The art of living consciously** |
|  | - 40 min sitting meditation - Present in the place you are - The 4 quadrants - The continuüm of anger | | | | | - Intention to give more love to… - Attention to seasons/environment |
| **Home-work** | - Daily meditation without CD - Noticing sensory experiences - Evaluation of the training | | | | |  |
| **8** | **And now onwards…** | | | | | **Mindfulness for the rest of your life** |
|  | - 40 min sitting meditation - Planning meditation after training - Living with attention - Formulating goals:   - 3 short term   - 3 long term | | | | | - Likert-scale feeling of being really alive - Evaluation of what you have learned - Short film of laughing people |
| **Follow-up online training Mindfulness for a healthier heart** | | | | | | |
| **Phase 2**  Four weeks continuing training | | **Week 9** | **Staying Mindful** | | | |
|  |  |  | 6-minute meditation practice  Planning mindfulness | | | |
|  |  | **Week 10** | **Rewrite your personal book of laws** | | | |
|  |  |  | What rules do you impose on yourself?  Which rules do you really have to adhere to?  What do you allow yourself to do? | | | |
|  |  | **Week 11** | **Mindful eating** | | | |
|  |  |  | Short film about eating in between daily chores  Assignment eating breakfast, lunch and dinner mindfully  Note your experiences | | | |
|  |  | **Week 12** | **Yoga for your heart** | | | |
|  |  |  | Yoga 30 minutes (film)  Likert-scale experiences  Planning Yoga into your schedule | | | |
| **Phase 3**.  Reminders every two weeks | | **Week 14** | **Rediscover your body** | | | |
|  |  |  | Go back to week 1, 3 websites with more meditation information | | | |
|  |  | **Week 16** | **Really listening** | | | |
|  |  |  | Go back to week 2 | | | |
|  |  | **Week 18** | **Stopping consciously** | | | |
|  |  |  | Go back to week 5, 4 websites with group training information | | | |
|  |  | **Week 20** | **Learning to meditate again** | | | |
|  |  |  | Go back to week 3, 8 book titles about mindfulness | | | |
|  |  | **Week 22** | **Planning Meditation** | | | |
|  |  |  | Go back to week 3, 3 CD tips with guided meditations | | | |
|  |  | **Week 24** | **Feeling alive** | | | |
|  |  |  | 5 likert scales | | | |
| **Phase 4**  Monthly reminders | | **Week 28** | **How do you deal with stress now?** | | | |
|  |  |  | Reflection on stressful situations and reactions, what would be a mindful reaction? | | | |
|  |  | **Week 32** | **Dealing with difficult thoughts** | | | |
|  |  |  | Reminder: when ruminating, consider facts | | | |
|  |  | **Week 37** | **Remember what you wanted to give more love?** | | | |
|  |  |  | 3 things you can do with love | | | |
|  |  | **Week 42** | **A beautiful quote** | | | |
|  |  |  | Reflection on Kabat-Zinn’s quote ‘human beings vs human doings’ | | | |
|  |  | **Week 46** | **Extra attention** | | | |
|  |  |  | Choose an activity to do more aware this week | | | |
|  |  | **Week 51** | **The feeling that you are alive** | | | |
|  |  |  | 5 Likert scales  Apps to continue practice | | | |

**Table B.** Regression model of training adherence (N=215)

|  | **β** | **SE** | **Standardized B** | **p-value** |
| --- | --- | --- | --- | --- |
| Intercept | -5.34 | 1.6 |  | 0.001 |
| Gender | 0.86 | 0.43 | 0.13 | 0.045 |
| Age | 0.03 | 0.02 | 0.16 | 0.084 |
| Diastolic Blood Pressure | 0.04 | 0.02 | 0.15 | 0.031 |

Dependent: LN (adherence%/1-adherence%), range -5 to 5.

**Table C.** Regression model of improvement on 6MWT when adherent (N=107).

|  | **β** | **SE** | **Standardized B** | **p-value** |
| --- | --- | --- | --- | --- |
| Intercept | -71.0 | 48.9 |  | 0.151 |
| Gender | -23.1 | 9.3 | -0.28 | 0.015 |
| BMI | -2.1 | 1.0 | -0.23 | 0.048 |
| Perceived stress | 2.6 | 0.9 | 2.77 | 0.007 |
| Mental QoL | 1.7 | 0.6 | 0.46 | 0.011 |

Dependent: Delta6MWT.
